# Entropy Generation Optimization in Multidomain Systems: A Generalized Gouy-Stodola Theorem and Optimal Control

**DOI:** 10.3390/e27060612

**Published:** 2025-06-09

**Authors:** Hanz Richter, Meysam Fathizadeh, Tyler Kaptain

**Affiliations:** Mechanical Engineering Department, Cleveland State University, Cleveland, OH 44115, USA; m.fathizadeh41@vikes.csuohio.edu (M.F.); t.kaptain@vikes.csuohio.edu (T.K.)

**Keywords:** energy optimization, entropy generation, multidomain systems, optimal control, electromechanical systems

## Abstract

The paper considers an extended interpretation of the second law of thermodynamics and its implications to power conversion optimization in multidomain systems. First, a generalized, domain-independent version of the classical Gouy-Stodola theorem is derived for interconnected systems which satisfy the Clausius postulate of the second law. Mechanical, electrical and more general Hamiltonian systems do not satisfy this postulate, however the related property of energy cyclodirectionality may be satisfied. A generalized version of the Gouy-Stodola theorem is then obtained in inequality form for systems satisfying this property. The result defines average forms of entropy generation and lost work for multi-domain systems. The paper then formulates an optimal control problem for a representative electromechanical system, obtaining complete, closed-form solutions for the load power transfer and energy harvesting cases. The results indicate that entropy generation minimization is akin to the maximum power transfer theorem. For the power harvesting case, closed-loop stability is guaranteed and practical controllers may be designed. The approach is compared against direct minimization of losses, both theoretically and with Monte Carlo simulations.

## 1. Introduction

Energy utilization improvement continues to be a dominant criterion for the design and operation of engineered systems. This motivation has become more pronounced in recent decades, as the environmental impact of power-consuming processes in transportation and manufacturing is understood and acknowledged. By 2050, the United States is projected to meet 80% of its electricity demand with renewable resources [1], however 60% of electricity was produced using fossil fuels in 2023 [2]. Many emerging technologies are incorporating electrification to either replace or supplement greenhouse-gas emitting devices. It is therefore important to understand the characteristics and limitations of power transmission in relation to system dynamics across various physical domains. Energy efficiency is an indicator of the amount of useful power produced at the output of a process relative to that required at the input. An economic cost, which may include an economic equivalent of environmental impact, is associated to power input, while a benefit is associated to power output, making efficiency maximization a desirable target. There exist situations, however, where the amount of power output is to be maximized, with less emphasis on efficiency. This situation may be encountered in energy harvesting from inexhaustible sources, such as ocean wave energy, where the operational cost of power input is zero.

Physical processes in which power from an input source is transferred to an external load must obey power conservation, which is prescribed by the First Law of Thermodynamics (FLT). This principle applies to all processes, regardless of the physical domains across which power is converted. The FLT places a constraint on the net power exchanged across system boundaries in relation to the rate of change of the energy stored within, so that the net sum is zero. But it does not restrict the allowable directions of power transfer. The Second Law of Thermodynamics (SLT) imposes such restriction. Several statements of the SLT were introduced in the 19th century as axiomatic principles. Clausius’ SLT *postulate* is that the heat (power) exchanged between two systems interacting only with each other may only flow from the hotter (more energetic) to the colder (less energetic) system. The widely-known Clausius inequality derives from this postulate.

### 1.1. Instantaneous Energy Directionality

Suppose that two systems 1 and 2 have stored energies $E_{1}\left(t\right)$ and $E_{2}\left(t\right)$, and that the power received by 1 from 2 is $\phi_{12}\left(t\right)$. Using energies instead of temperatures, Clausius’ SLT statement can be summarized as

(1) $$\phi_{12}\left(t\right)\left(E_{2}\left(t\right)-E_{1}\left(t\right)\right)\geq 0$$

for all *t*, with $\phi_{12}\left(t\right)=0$ if and only if $E_{1}\left(t\right)=E_{2}\left(t\right)$.

Like the FLT, the SLT is thought to apply to all physical systems [3]. However, an important class of systems, which includes the lumped-parameter models of mechanical and electrical systems commonly used in engineering, do not verify the above *at each instant of time*. It is straightforward to obtain counterexamples with simple mechanical systems or circuits, see for instance [4]. The instantaneous power across mechanical and electrical (and the more general class of Hamiltonian systems) may flow “backwards”, that is, from low- to high-energy subsystems at particular instants of time.

The inability of these models to verify the SLT is arguably connected to their macroscopic, lumped-element nature. The SLT can be derived from statistical mechanics, which derives ensemble properties from a large group of microscopic particles. Because each particle obeys Hamiltonian mechanics, the SLT assigns a very small probability, but does not totally rule out processes violating Clausius’ statement.

Attempts to reconcile the SLT with the macroscopic models conventionally used for mechanical systems and electric circuits were made in the early 20th century starting with the work of Onsager [5] and developed further by Meixner [6] and Flower [7]. These works considered linear phenomenological laws for circuit elements and isothermal conditions, and focused on structural properties and their relation to stability and reversibility, but do not offer a complete picture of their to compliance with the SLT. Further, researchers have attempted to ascribe entropy production to electric circuits by considering isothermal operation at $T_{o}$ or between fixed-temperature baths [7,8,9,10]. They assumed that circuits were able to instantaneously carry all dissipation heat away, so that their temperature remained constant. The time-domain energy-balance is then used with a normalization factor of $T_{o}$ to define entropy generation.

The lumped-parameter models used widely in engineering to describe the behavior of mechanical, electrical and coupled systems do not include temperature among the participating variables. This presents and obstacle to meaningful thermodynamic interpretations of system behavior, particularly concerning the SLT, given that classical thermodynamics defines entropy on the basis of temperature. As discussed below, recent formalizations of thermodynamics under a dynamical systems perspective replace temperatures with energies, recovering SLT principles in a more general setting. Such approach is adopted in this paper.

By far, the vast amount of work across thermodynamics and control is concerned with thermodynamic systems in the classical sense, and includes the notion of temperature [11,12,13] or uses control to impose unidirectional power transfer or thermal-like behavior [14,15,16]. Recently, ectropy (a quadratic function of energy) was used for stabilization and maximum power point tracking in generators used in wind power [17,18]. But ectropy was used for its ability to define a control Lyapunov function rather than to analyze the energy behavior of the system.

### 1.2. Extended Interpretations of the SLT and Cyclo-Dissipativity

The question of whether the SLT and its implications can be evidenced in extended physical domains has generated recent interest among researchers in the dynamics and controls field. Also, a comprehensive understanding of thermodynamic principles in the context of dynamical systems theory was developed in the recent years by Haddad and co-workers [19,20,21]. That work pertains to interconnected dynamic systems that satisfy the Clausius statement above. Upon this axiomatic basis, the work defines and links entropy and the principle of entropy non-increase for isolated systems to storage functions in dissipative systems theory.

More recently, van der Schaft [22] developed a dynamical theory for thermodynamic systems on the basis of the Kelvin postulate, which states that it is impossible for a process operating cyclically to produce positive work while interacting with a single thermal reservoir at constant temperature. Kelvin’s postulate can be framed using the concept of *cyclo-dissipativity* [23,24]. A system having input *u* and output *y* said to be cyclopassive with respect to the supply rate s(u,y) if ∮s(u(t),y(t))dt≥0 for all periodic trajectories. Kelvin’s postulate involves the rate of volumetric expansion work as a supply rate −PV˙, where *P* is the pressure and V the volume. Further, a study by the same author [25] considers whether systems outside thermodynamics are subject to a similar restriction. It was shown that the fairly general class of port-Hamiltonian systems may be so restricted under a sufficient condition given in the work.

**Figure 1:**
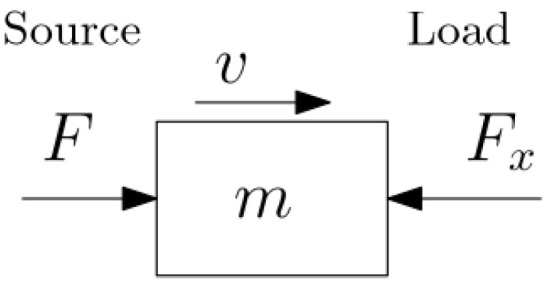
Performing Work Across a Mass.

To illustrate this aspect, consider two forces *F* and $F_{x}$ acting in opposite directions on a mass with velocity *v* as in Figure 1. To maintain correspondence with thermodynamics and heat engines, suppose that Fv represents power drawn from a source and $F_{x}v$ represents power transmitted to a load, and assume motion is periodic with period *T*. Per Newton’s law $m\dot{v}=F-F_{x}$, and the FLT is seen by multiplying each side by *v*:

$$\dot{K}=\frac{d}{dt}\frac{1}{2}mv^{2}=m\dot{v}v=Fv-F_{x}v$$

where *K* is the kinetic energy and the right-hand side is the net power exchanged with the source and load. In Kelvin’s statement, *F* is constant and motion is cyclic, requiring v(0)=v(T). Integrating

$$0=\int_{0}^{T}F_{x}v\;dt=W$$

which shows that no work *W* may be done in this manner, even without frictional dissipation. The above system is *one-port cyclopassive* with respect to either source or load ports. With friction, W≤0 is obtained. Several examples of multidomain systems complying or not complying with the SLT in this sense are provided in [25].

### 1.3. Entropy Generation Minimization and the Gouy-Stodola Theorem

The above corresponds to a *total* impossibility to perform useful work, and under the condition that one of the input variables defining source power is constant. This has the shortcoming of not describing the limitations to power conversion that may arise when the latter condition is not met. The limitation in this case is not binary and must be quantified, for instance through efficiency, as thoroughly described in classical thermodynamics. A fundamental limitation to efficiency comes from the FLT and the fact that losses to the environment are always non-negative. Further, the SLT provides an upper bound to efficiency, as in the maximal efficiency of a reversible heat engine derived by Carnot. Carnot efficiency may be reached only by idealized processes involving heat transfer across infinitesimal temperature differences, unrestricted volumetric expansion and state changes along an equilibrium manifold.

#### Entropy Generation

Classical thermodynamics defines entropy *S* as a state function, a quantity whose change ΔS between two states is independent of the path followed. Clausius defined entropy generation (also known as energy production) for a closed system as the sum of two parts:

(2) $$\Delta S=S_{gen}+\oint \frac{\delta Q}{T}$$

where *Q* is the heat power exchanged between the system and the surroundings, *T* is the system’s temperature and $S_{gen}$ is the entropy production. The δ notation is used to distinguish the infinitesimal variation of quantities which are not state functions, whereas the differential notation is used for state functions.

Clausius inequality ∮δQ/T≤ΔS then implies that $S_{gen}\geq 0$ for all processes, with equality corresponding to reversible ones. Entropy generation is frequently presented in rate form:

(3) $$\dot{S}={\dot{S}}_{gen}+\frac{\delta Q}{T}$$

Entropy generation was linked to the degree of non-ideality of a process and to the sub-maximality of the work produced by a system. The Gouy-Stodola theorem, originally stated in 1889 [26] and subsequently reconsidered in various settings [27] establishes that the difference between the work obtained under ideal conditions and the actual work produced, termed *lost work*, $W_{l}$ is given by

(4) $$W_{l}=T_{0}S_{gen}$$

where $T_{0}$ is the constant temperature of the surroundings.

To account for the conditions of non-ideal processes, the concept of *entropy generation minimization* (EGM) was introduced by Bejan [28] as a criterion to optimize thermodynamic systems. The method is based on the Gouy-Stodola theorem and traditionally applied to classical thermodynamic systems.

This paper provides a self-contained description of entropy generation and a generalization of the Gouy-Stodola theorem for multidomain systems. To the best of the authors’ knowledge, this is the first generalization of this kind. For the classical thermodynamics definition of entropy generation, refer for instance to [28].

EGM may be used for component selection or parameter optimization, as mostly done in the original work of Bejan. To a limited extent, entropy generation or the related exergy have also been used as objective functions in optimal control problems [29,30], but always for thermodynamic systems. It has been shown [31] that irreversibility minimization, efficiency maximization and power maximization all produce different optimal solutions for thermodynamic systems. This paper considers power transmission optimization problem for multidomain systems from the vantage point of the SLT.

Previous work by the authors [4] showed that Clausius’ statement may apply to a wide class of multidomain systems if only cyclic processes are considered. The corresponding average subsystem energies are used in place of temperatures, along with the average power transmission across subsystems. This was regarded as a property of systems rather than an axiomatic principle, and referred to as Energy Cyclo-Directionality (ECD). The cited work shows that for linear systems, ECD can be established by solving a generalized eigenvalue problem. Subsequently, the property has been examined for nonlinear systems such as robotic manipulators [32]. ECD provides a way to quantify efficiency in multidomain systems by paralleling thermodynamics, in particular using average entropy generation and a generalized interpretation of the Gouy-Stodola theorem. The approach was successfully used in [33] as part of an $H_{\infty }$ control problem to obtain disturbance rejection together with disturbance energy harvesting.

The main contributions of this paper are: i. a description of entropy generation in generalized interconnected thermodynamic systems (i.e., those obeying Clausius’ statement) and interconnected multidomain systems satisfying ECD, resulting in a generalized version of the Gouy-Stodola theorem, ii. the formulation, solution and complete characterization of optimal control problems based on EGM for a representative electromechanical system, for load power transfer and energy harvesting cases, and iii: a theoretical and numerical comparison between the EGM-based controllers and traditional efficiency maximization. As part of iii., a connection between EGM and the maximum power transfer theorem is made, along with an elucidation of the compromise between stability and the direction of average power transfer.

The remainder of the paper is organized as follows: Section 2 provides a modeling framework and power balances, Section 3 introduces entropy generation and derives the Gouy-Stodola theorem for interconnected generalized thermodynamic systems, Section 4 does the same for multidomain systems, Section 5 formulates and solves and optimal control problem for an electromechanical system based on entropy generation and examines solution properties and efficiency, and finally Section 7 offers concluding remarks and topics for further investigation.

## 2. Interconnected System and Average Power Balances

**Figure 2:**
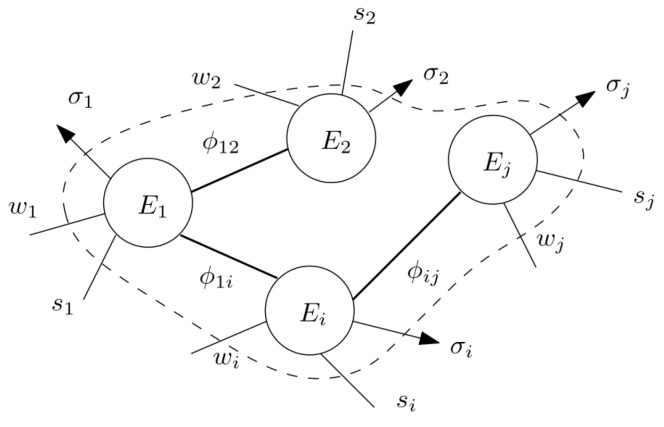
Interconnection of Energy-Storing Subsystems.

We consider an interconnection of *N* energy-storing subsystems as in Figure 2. The instantaneous power exchanged between two subsystems is denoted as $\phi_{ij}$, with the sign convention that $\phi_{ij}>0$ corresponds to power transfer from *j* to *i*. Thus $\phi_{ij}=-\phi_{ji}$. Subsystems transfer power with each other through $\phi_{ij}$ and with the exterior through three categories of power defined as follows: first, $s_{i}$ are power supplies in the usual sense, where $s_{i}>0$ indicates power added to subsystem *i* and $s_{i}<0$ indicates “regenerative” power returned to the source; $w_{i}>0$ denotes the rate of work done by *i* on the environment, again allowing for $w_{i}<0$, and the third category corresponds to power dissipation $\sigma_{i}$, which is non-negative and shown with arrows in Figure 2.

The interconnected system is assumed to be a dynamic system with state *x*, inputs *u* and outputs y(x,u). The energies stored in each subsystem are assumed to be non-negative functions $E_{i}\left(x,u\right)$, and $s_{i}$, $\phi_{ij}$, $w_{i}$ and $\sigma_{i}$ are all functions of *y*, *u* and possibly explicitly dependent on time *t*.

At every instant, the FLT requires that a power balance must hold for each *i*:

(5) $${\dot{E}}_{i}=s_{i}+\sum_{j=1,j\neq i}^{N}\phi_{ij}-\sigma_{i}-w_{i}$$

where arguments have been omitted.

### Cyclic Trajectories

We use the cyclic integral notation ∮ with or without displaying the limits to refer to integration over cyclic trajectories over the time interval [0T], i.e., x(0)=x(T) and u(0)=u(T). For such trajectories, all power functions above are themselves cyclic, and in particular $\oint {\dot{E}}_{i}\left(x\left(t\right),u\left(t\right)\right)dt=\oint dE_{i}=0$. Then an *average power balance* below holds for each *i*:

(6) $$0={\bar{s}}_{i}+\sum_{j=1,j\neq i}^{N}{\bar{\phi }}_{ij}-{\bar{\sigma }}_{i}-{\bar{w}}_{i}$$

where ${\bar{s}}_{i}=\frac{1}{T}\int_{0}^{T}s\left(y\left(t\right),u\left(t\right),t\right)dt$ and similar definitions apply to ${\bar{\phi }}_{ij}$, ${\bar{\sigma }}_{i}$ and ${\bar{w}}_{i}$. Summing over *i*, an average power balance is obtained for the whole system:

(7) $$0=\bar{S}-\bar{\sigma }-\bar{W}$$

where all ϕ terms cancel and $\bar{S}=\sum_{i=1}^{N}{\bar{s}}_{i}$, $\bar{W}=\sum_{i=1}^{N}{\bar{w}}_{i}$ and $\bar{\sigma }=\sum_{i=1}^{N}{\bar{\sigma }}_{i}$. This represents power conservation, which is the same as the FLT for the interconnected system.

## 3. Generalized Thermodynamic Systems

This section is a bridge between classical thermodynamics and the intended generalization of SLT principles to multidomain systems. The energies of the interconnected subsystems are used instead of temperatures and an energy-based version of Clausius’ postulate is assumed to hold at each instant of time. We formally recover the Gouy-Stodola theorem in this setting.

In thermodynamics, *s* represents the rate of heat received by the system from the environment, while σ represents the rate of heat rejected by the system to the environment. For cyclic trajectories, define the net heat associated with subsystem *i* as

(8) $${\bar{\delta q}}_{i}={\bar{s}}_{i}-{\bar{\sigma }}_{i}$$

with this definition, Equation (7) adopts the form commonly presented in thermodynamics for closed systems (no mass transfer across boundaries) and cyclic processes:

(9) $$\bar{\delta Q}=\bar{W}$$

where $\bar{\delta Q}=\sum_{i=1}^{N}{\bar{\delta q}}_{i}$.

### 3.1. Directionality Axiom

In a thermodynamics setting, $\phi_{ij}$ represents a heat flow interaction, and subsystem temperatures are an indication of stored energy. For ideal gases, energy is $E=C_{v}\left(T\right)T$, indeed a function of temperature alone. The axiom below [19] models the physical fact that heat may only flow from high temperatures to low temperatures (or more generally, energies), with no heat transfer occuring upon temperature equality:

(10) $$\begin{array}{ccc} \left(E_{i}\left(t\right)-E_{j}\left(t\right)\right)\phi_{ji}\left(t\right) & \geq & 0 \end{array}$$

(11) $$\begin{array}{ccc} \phi_{ji}\left(t\right)=0 & \Leftrightarrow & E_{j}\left(t\right)=E_{i}\left(t\right) \end{array}$$

for all t≥0. In a slightly more general form, the axiom is presented with scaled and shifted energies:

(12) $$\left(d_{i}\left(t\right)-d_{j}\left(t\right)\right)\phi_{ji}\left(t\right)\geq 0$$

where $d_{i}\left(t\right)=\beta_{i}E_{i}\left(t\right)+c$, for some nonnegative constants *c*, $\beta_{i}$ and $\beta_{j}$. This axiom is used in the derivations of this section, pertaining generalized thermodynamic systems. It will be replaced by an averaged version known as energy cyclo-directionality for multidomain systems in Section 4 and thereafter.

### 3.2. Thermodynamically-Consistent Work

In this section, we define $w_{i}$ as *thermodynamically-consistent work* if it satisfies the following property for all *i*:

(13) $$\oint \frac{w_{i}}{d_{i}}dt=0$$

for all cyclic trajectories. This definition is modeled after the classical volumetric expansion work of the form w=PV˙ where V is the volume and *P* the pressure, which is consistent per this definition. Indeed, for a pure substance, using E=u(s,V) as the internal energy in terms of independent variables *s* (entropy) and V, Gibbs’ relationship defines the pressure as

P=−∂u(s,V)∂V

and

du=∂u(s,V)∂sds−PdV

Using d=βu+c to divide through and integrate over a cycle:

0=∮duβu+c=∮1βu+c∂u(s,V)∂sds−∮PdVβu+c

Equation (13) follows because *s* and V are independent, and for a cycle:

∮1βu+c∂u(s,V)∂sds=lnβu(s(T),V(T))+cβu(s(0),V(0))+c=0

### 3.3. Average Entropy Generation and Clausius Inequality

The instantaneous power balance of Equation (5) contains $s_{i}-\sigma_{i}$, which in thermodynamics is the net heat flow $\delta q_{i}$ into the subsystem. Entropy is defined as $\delta q_{i}/T_{i}$. To evidence entropy-like features of the interconnected system, divide each equation through $d_{i}$ to obtain

(14) $$\frac{{\dot{E}}_{i}}{d_{i}}=\frac{\delta q_{i}}{d_{i}}+\frac{1}{d_{i}}\sum_{j=1,j\neq i}^{N}\phi_{ij}-\frac{w_{i}}{d_{i}}$$

Assuming that $w_{i}\left(y,u,t\right)$ is assumed to be thermodynamically consistent, cyclic integration gives

(15) $$0=\oint \frac{\delta q_{i}}{d_{i}}dt+\oint \frac{1}{d_{i}}\sum_{j=1,j\neq i}^{N}\phi_{ij}\;dt$$

Summing across all subsystems:

(16) $$0=\oint \sum_{i=1}^{N}\frac{\delta q_{i}}{d_{i}}dt+{\bar{X}}_{g}$$

where the *average entropy generation* or *irreversible entropy production* [22] is

(17) $${\bar{X}}_{g}=\oint \sum_{i=1}^{N}\sum_{j=1,j\neq i}^{N}\frac{\phi_{ji}\left(d_{i}-d_{j}\right)}{d_{i}d_{j}}\;dt$$

For generalized thermodynamic systems, the directionality axiom of Equation (12) is adopted, showing that ${\bar{X}}_{g}\geq 0$, and thus

(18) $$\oint \sum_{i=1}^{N}\frac{\delta q_{i}}{d_{i}}dt\leq 0$$

which is *Clausius inequality* for the interconnected system. Note that the directionality axiom as stated holds for every instant of time. Note also that Clausius inequality would continue to hold if the integral in Equation (17) is nonnegative for all cyclic trajectories, which is a weaker condition. The inequality becomes an equality only whenever ${\bar{X}}_{g}=0$. This condition can be connected to the possibility of reversing the cycle so that both the system and the environment return to their original states [19].

### 3.4. The Gouy-Stodola Theorem for Interconnected Systems

A limitation of the total system’s ability to perform work on the exterior follows directly from Clausius inequality and the FLT for the entire system. The Gouy-Stodola Theorem is re-derived in the context of this paper to facilitate the generalization in Section 4.

Note that Clausius inequality does not contain work terms and depends on *N* quantities $\delta q_{i}$, which are constrained through the instantaneous power balance for the total system: $\sum \delta q_{i}=\sum w_{i}≜W$. This can be used to replace one arbitrarily-selected $\delta q_{k}$ and introduce a term containing *W*. Let *k* denote the so-chosen *reference*, and use

(19) $$\delta q_{k}=W-\sum_{i=1,i\neq k}^{N}\delta q_{i}$$

Substitute this into Clausius inequality and rearrange to obtain

(20) $$\oint \frac{W}{d_{k}}dt\leq \oint \sum_{i=1,i\neq k}^{N}\delta q_{i}\left(\frac{1}{d_{k}}-\frac{1}{d_{i}}\right)\;dt$$

The classical Gouy-Stodola theorem holds for a constant-temperature environmental reference. In this paper this corresponds to assuming $d_{k}$ constant through the cycle, so that

(21) $$\bar{W}\leq \oint \sum_{i=1,i\neq k}^{N}\delta q_{i}\left(1-\frac{d_{k}}{d_{i}}\right)\;dt≜{\bar{W}}_{max}$$

Define *lost work* as ${\bar{W}}_{l}≜{\bar{W}}_{max}-\bar{W}$ and perform the subtraction to obtain:

(22) $${\bar{W}}_{l}=\oint \sum_{i=1,i\neq k}^{N}\delta q_{i}\left(1-\frac{d_{k}}{d_{i}}\right)-\sum_{i=1}^{N}\delta q_{i}=-d_{k}\oint \sum_{i=1}^{N}\frac{\delta q_{i}}{d_{i}}\;dt$$

but from Equation (16) this reduces to

(23) $${\bar{W}}_{l}=d_{k}{\bar{X}}_{g}$$

which generalizes Gouy-Stodola’s result for an interconnected thermodynamic system considering cyclic trajectories. Note that ${\bar{W}}_{max}$ is not an absolute quantity; it is relative to the total energies stored in [0T] and the net heat exchanges with the environment in the same period. Cycles were considered to maintain correspondence with the results below, but note that the same result is reached without the periodicity assumption, due to the directionality axiom holding at each *t*.

### 3.5. Other Forms of Work

To generalize the above properties into other domains, more general forms of work must be included, that is $w_{i}$ may not satisfy Equation (13). For this purpose, we now consider that there are two components of work rate:

(24) $$w_{i}=w_{ri}+w_{oi}$$

where only $w_{ri}$ is thermodynamically-consistent. It is direct to see that if we redefine $\delta q_{i}$ to include $w_{oi}$, namely $\delta q_{i}=s_{i}-\sigma_{i}-w_{oi}$, then Clausius inequality is obtained exactly as before. However, this form of energy accounting would not allow us to keep track of the total work done by the $w_{oi}$ components, since the term would be considered as part of the “net heat”. Further analysis shows that an inequality form of Gouy-Stodola’s theorem still holds when $w_{oi}$ are present. This requires an additional assumption:

**Assumption** **1.** *There exists a finite constant $\hat{L}$ such that*

(25) $$\oint \sum_{i=1}^{N}\frac{w_{oi}}{d_{i}}\;dt\leq \hat{L}$$

*for all cyclic trajectories.*

As shown in Section 5 an explicit value of $\hat{L}$ may be found by considering the specific system dynamics. However, the value of $\hat{L}$ is not needed in what follows. As before, divide each equation in the power balance by the corresponding $d_{i}$ and integrate cyclically to obtain

(26) $$\oint \sum_{i=1}^{N}\frac{{\dot{E}}_{i}}{d_{i}}\;dt=0=\oint \sum_{i=1}^{N}\frac{\delta q_{i}}{d_{i}}\;dt+{\bar{X}}_{g}-\oint \sum_{i=1}^{N}\frac{w_{oi}}{d_{i}}\;dt$$

Using the directionality axiom, ${\bar{X}}_{g}\geq 0$ and

(27) $$\oint \sum_{i=1}^{N}\frac{\delta q_{i}}{d_{i}}\leq \sum_{i=1}^{N}\frac{w_{oi}}{d_{i}}\leq \hat{L}$$

Now consider the ends of the above inequality and as before eliminate a $\delta q_{k}$ to favor *W*. Assuming again that $d_{k}$ is constant, the following holds:

(28) $$\bar{W}\leq d_{k}\hat{L}+\oint \sum_{i=1,i\neq k}^{N}\delta q_{i}\left(1-\frac{d_{k}}{d_{i}}\right)\;dt≜{\bar{W}}_{max}$$

Subtracting $\bar{W}$ from ${\bar{W}}_{max}$ and simplifying:

(29) $${\bar{W}}_{l}={\bar{W}}_{max}-\bar{W}=d_{k}\left(L+{\bar{X}}_{g}dt-\sum_{i=1}^{N}\frac{w_{oi}}{d_{i}}\right)$$

But from Assumption 1 we have

(30) $$-d_{k}\sum_{i=1}^{N}\frac{w_{oi}}{d_{i}}\leq d_{k}\hat{L}$$

and a more general version of the Gouy-Stodola Theorem is obtained in inequality form:

(31) $${\bar{W}}_{l}\geq d_{k}{\bar{X}}_{g}$$

## 4. Multidomain Systems

The challenges to generalize the above “lost work” characterizations to extended physical domains are connected to the directionality axiom used for thermodynamic systems. Indeed, the axiom does not hold for mechanical, electrical and magnetically-coupled electromechanical systems, because *instantaneous* power transfer patterns between subsystems in relation to their relative energies do not follow those of heat flow. Simple examples showing this can be consulted in [4]. However, a property similar to the inequality of the axiom can be established for these systems by considering only cyclic trajectories and the corresponding energy averages in scaled form (and shifted with *c* if necessary). To facilitate subsequent developments, all periodic trajectories are assumed to be in the signal space $L_{2}\left[0\;T\right]$ of square-integrable functions, defined by

$$L_{2}\left[0\;T\right]={\left\{f:||f|\right|}_{2}^{2}≜\int_{0}^{T}f^{2}\left(t\right)dt<\infty \}$$

where the signal 2-norm has been defined for functions f:R↦R. The notation P is used to refer to a given subset of such periodic trajectories.

### 4.1. Energy Cyclo-Directionality Property (ECD)

Multidomain systems fail to verify the energy directionality axiom reflecting Clausius postulate, precluding results such as the generalized Gouy-Stodola theorem of Equation (31). Recently, it was observed that a property similar to Equation (10) may hold if only cyclic trajectories are considered and average quantities are used instead of instantaneous ones.

The interconnected system is said to satisfy energy cyclo-directionality (ECD) for trajectories in P if there are constants $\beta_{i,j}\geq 0$ such that

(32) $$\left(\beta_{i}{\bar{E}}_{i}-\beta_{j}{\bar{E}}_{j}\right){\bar{\phi }}_{ji}\geq 0$$

for all trajectories in P. Research by the authors investigated methods to verify the ECD property and to obtain the β constants for a given interconnected system. For linear systems, the calculation was shown in [4,33] to reduce to the solution of a generalized eigenvalue problem, and examples were provided. The verification of ECD for nonlinear mechanical systems such as robotic manipulators is considered in [32]. Here we discuss how ECD may be established for three representative linear and nonlinear systems.

### 4.2. ECD for Three Example Systems

**Figure 3:**
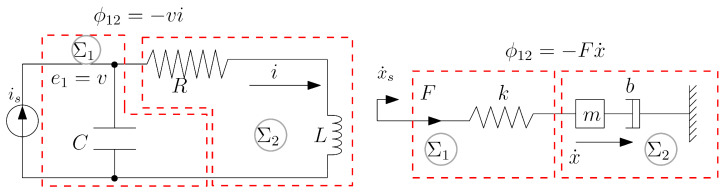
RLC circuit with current source and its mechanical analog.

**Linear RLC circuit and mechanical analog**: The systems in Figure 3 have the same dynamics after mapping mass velocity to current and spring deformation to capacitor charge. Two subsystems have been defined according to the indicated boundaries. In each domain, subsystem 2 is cyclopassive. Indeed, using the circuit as an example, the mass and damper can only dissipate power over cyclic motions, thus ${\bar{\phi }}_{21}\geq 0$. Then ECD reduces to finding γ such that ${\bar{E}}_{2}-\gamma {\bar{E}}_{1}\leq 0$ for all cyclic trajectories. The average energies are quadratic functions of frequency and the ECD can be established by finding $\gamma_{crit}$ in

$$\gamma_{crit}=\underset{\omega }{\sup}\frac{{\bar{E}}_{2}\left(\omega \right)}{{\bar{E}}_{1}\left(\omega \right)}$$

An analytical solution is obtained as $\gamma_{crit}=\frac{L}{CR^{2}}$ (or analogously $\gamma \geq \frac{mk}{b^{2}}$ for the mechanical system).

**Figure 4:**
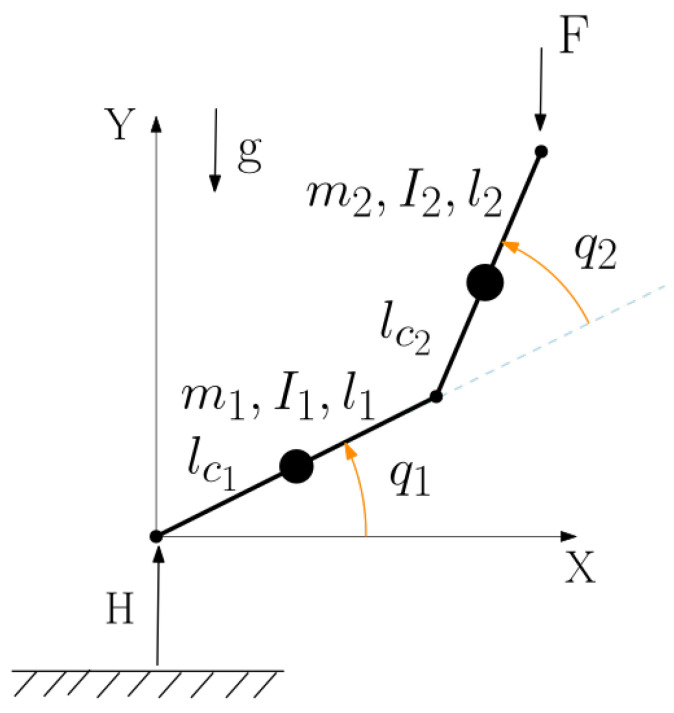
Two-link robotic arm.

**Two-link robotic manipulator**: consider the system of Figure 4.

Two subsystems may be defined so that each contains the total energy of a link (kinetic plus potential) and $\phi_{12}$ is the power transmitted between them. The instantaneous power balance is

(33) $$\begin{array}{c} {\dot{E}}_{1}={\dot{K}}_{1}+{\dot{V}}_{1}=\phi_{12}+\tau_{1}\left(t\right){\dot{q}}_{1}\left(t\right)\\ {\dot{E}}_{2}={\dot{K}}_{2}+{\dot{V}}_{2}=\phi_{21}+\tau_{2}\left(t\right){\dot{q}}_{2}\left(t\right) \end{array}$$

where $K_{i}$ and $V_{i}$ are the potential and kinetic energies of link *i* and $\tau_{i}$ is the input torque at joint *i*. For periodic motion and in the absence of dissipation we have

(34) $$\begin{array}{c} 0={\bar{\phi }}_{12}+\oint \tau_{1}\left(t\right){\dot{q}}_{1}\left(t\right)\;dt\to {\bar{\phi }}_{12}=-\oint \tau_{1}\left(t\right){\dot{q}}_{1}\left(t\right)\;dt \end{array}$$

Unlike the previous example, the sign of ${\bar{\phi }}_{12}$ may change depending on the specific cyclic trajectory. Restricting the class of trajectories P to those yielding ${\bar{\phi }}_{12}$ of a definite sign, ECD may be satisfied by considering an inequality on the weighted average energies alone, as follows: if ${\bar{\phi }}_{12}<0$, enforce $(\gamma_{12}{\bar{E}}_{1}-{\bar{E}}_{2})>0$, or equivalently $({\bar{E}}_{2}-\gamma_{12}{\bar{E}}_{1})<0$. Conversely, if ${\bar{\phi }}_{12}>0$, enforce $({\bar{E}}_{1}-\gamma_{21}{\bar{E}}_{2})<0$. In either case, γ can be found from

(35) $$\gamma_{ij}=\underset{P}{\sup}\;\;\frac{{\bar{E}}_{j}}{{\bar{E}}_{i}}$$

For the case ${\bar{\phi }}_{12}>0$ the problem becomes the maximization of function *J* below:

(36) $$J\left(q_{1},{\dot{q}}_{1},q_{2},{\dot{q}}_{2}\right)=\frac{{\bar{E}}_{1}}{{\bar{E}}_{2}}=\frac{\frac{1}{T}\oint E_{1}dt}{\frac{1}{T}\oint E_{2}dt}$$

The problem is solvable analytically with calculus of variations techniques. In [34] it is shown that if the potential energy reference level is shifted to a high-enough value, γ is found at a static configuration where the links are both downright.

**Figure 5:**
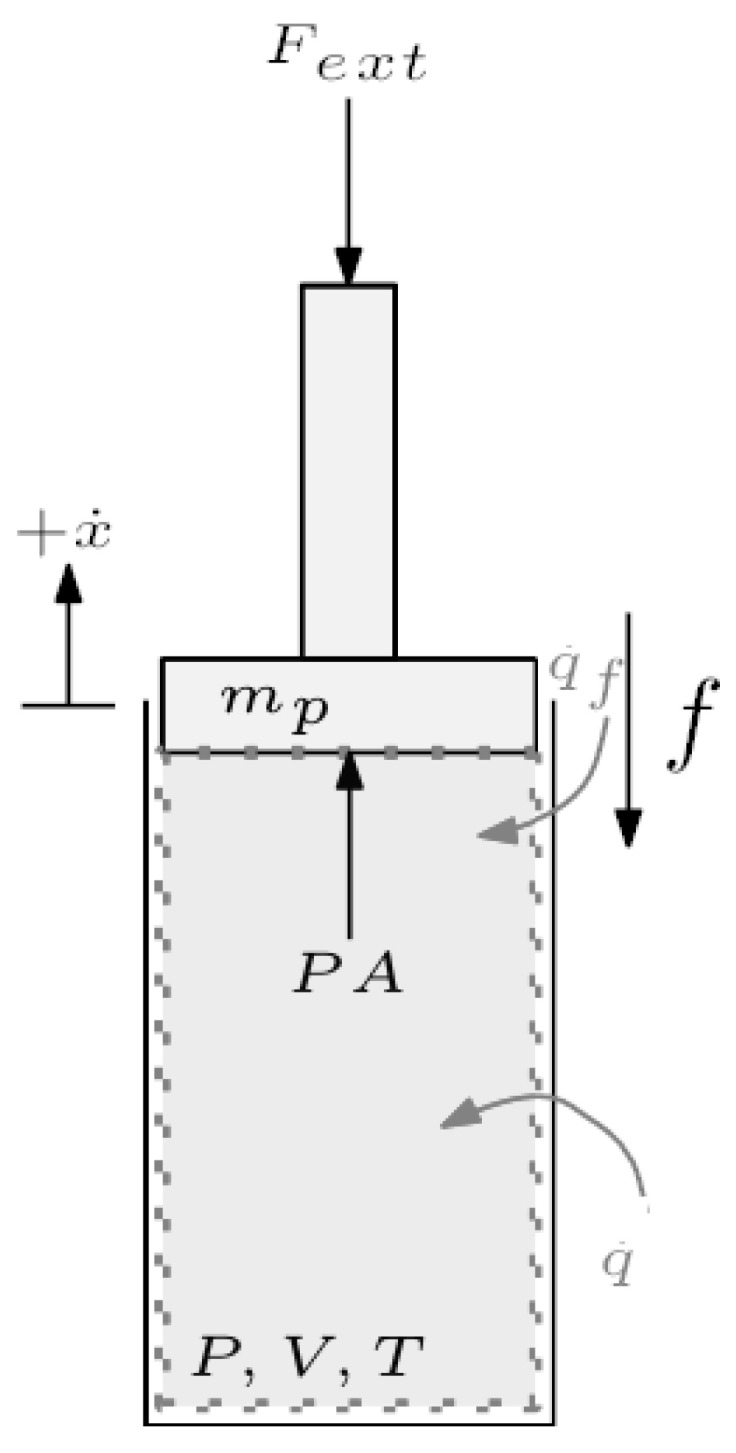
Piston-Mass System.

**Thermo-mechanical system**: Consider a cylinder-piston system with an ideal gas and a heat source *q* (with units of power). The piston is assumed to have mass *m* and a frictional force *f* between the piston and the cylinder walls produces heat $q_{f}$ that is all directed to the gas. The system performs work against an external force $F_{ext}$ as in Figure 5. Assume that there is viscous friction in the mechanical system with coefficient $b_{m}$. With states defined as piston position $x_{1}$ and velocity $x_{2}$ together with gas temperature $x_{3}$, system dynamics become ${\dot{x}}_{1}=x_{2}$ together with

(37) $$\begin{array}{ccc} m_{g}C_{v}{\dot{x}}_{3} & = & q+fx_{2}-m_{g}R_{g}x_{2}\frac{x_{3}}{x_{1}} \end{array}$$

(38) $$\begin{array}{ccc} J{\dot{x}}_{2} & = & m_{g}R_{g}\frac{x_{3}}{x_{1}}-F-f-b_{m}x_{2} \end{array}$$

where $C_{v}$ is the heat capacity of the gas at constant volume and $R_{g}$ is the universal ideal gas constant. Define subsystems g and m to correspond to energy storage in the ideal gas (as internal energy) and in the mass (as kinetic energy). The instantaneous power balance is obtained by multiplying Equation (38) by $x_{2}$ and noting that Equation (37) is already a power balance, since the energy stored in the gas is $E_{g}=m_{g}C_{v}x_{3}$. Then the terms participating in the power balance of Equation (5) are identified for the gas as $s_{g}=q$, ϕgm=−PV˙+fx˙=mgRgx3x2/x1−bcx22, $\sigma_{g}=0$ and $w_{g}=0$. For the mechanical subsystem, $s_{m}=0$, $\sigma_{m}=b_{m}{\dot{x}}_{2}^{2}$, $w_{m}=F_{ext}x_{2}$ and $\phi_{mg}=-\phi_{gm}$.

Over periodic trajectories, the average quantities are defined by integration and the average balance of Equation (6) is obtained. As in the previous example, the sign of ${\bar{\phi }}_{mg}$ is not definite and the class of periodic trajectories must be restricted to facilitate the verification of ECD. If the system is intended to produce work from heat, we may define P as the set of periodic trajectories for which ${\bar{\phi }}_{mg}>0$. In this case ECD reduces to finding γ so that

$$\gamma {\bar{E}}_{g}-{\bar{E}}_{m}\geq 0$$

for all trajectories in P, which is equivalent to finding $\gamma_{crit}$ as

$$\gamma_{crit}=\underset{P}{\sup}\frac{{\bar{E}}_{m}}{{\bar{E}}_{g}}$$

and taking $\gamma \geq \gamma_{crit}$ to satisfy ECD. This problem must be solved by numerical optimization.

### 4.3. The Gouy-Stodola Theorem for Multidomain Systems

In the previous sections, division by $d_{i}$ was used first, followed by cyclic integration, as in thermodynamics, and the directionality axiom of Equation (10) leads to non-negativity of ${\bar{X}}_{g}$. The approach of the following work is to integrate first and then divide, so that the ECD property can be used to define a non-negative average entropy generation, reaching the same conclusions regarding lost work as in thermodynamics. For multidomain systems there is no need or benefit to consider that a thermodynamically-consistent work component exists. This is because Equation (13) does not imply that $\sum \frac{\oint w_{oi}\;dt}{d_{i}}=0$. Only a general work term $w_{i}$ will be used.

A quantity analogous to $X_{g}$ above arises when each equation in the average power balance of Equation (6) is divided by the corresponding $d_{i}$ and all equations added, to obtain:

(39) $$\sum_{i=1}^{N}\frac{\delta {\bar{q}}_{i}}{d_{i}}+{\bar{X}}_{g}-\sum_{i=1}^{N}\frac{{\bar{w}}_{i}}{d_{i}}=0$$

where $\delta {\bar{q}}_{i}={\bar{q}}_{i}-{\bar{\sigma }}_{i}$. The ECD property implies that ${\bar{X}}_{g}\geq 0$. A counterpart to Assumption 1 is now stated as follows:

**Assumption** **2.** *The interconnected system satisfies*

$$\sum_{i=1}^{N}\frac{{\bar{w}}_{i}}{d_{i}}\leq \hat{L}$$

*for some finite constant $\hat{L}$ and all periodic trajectories.*

The verification of this assumption must be done in consideration to particular system dynamics. In this paper, the assumption is satisfied for the closed-loop system resulting from the solution of the optimal control problems in Section 5. The formal steps to obtain the inequality form of the Gouy-Stodola Theorem are exactly the same as for thermodynamic systems, noting that now $d_{k}$ is indeed constant for a given cyclic trajectory. The result is then stated as

(40) $${\bar{W}}_{l}\geq d_{k}{\bar{X}}_{g}$$

where the lost work is now an average quantity over a periodic trajectory.

Equation (40), based on ECD and Assumption 2, justifies the use of EGM for multidomain systems. As with any other optimization scenario, appropriate boundary conditions and constraints must be imposed. For lost work minimization, a reference $d_{k}$ must be selected. The question of the effect of this choice on the outcomes of optimization remains under study, but the results of the following sections are based on a choice which simplifies the cost function. A similar situation occurs in exergy-based thermodynamic optimization. Research on classical irreversible thermodynamics shows that there is some disagreement on the appropriate choice of a reference, in particular when its properties may not be constant [35].

## 5. Optimal Control of an Electromechanical System

We consider an electromechanical system known as a voice-coil actuator (VCA). The problem is cast as lost work minimization under the periodic trajectories setting of this paper. Two separate cases are considered: A: EGM-based enhancement of power transfer to the mechanical port, and B: EGM-based enhancement of power harvested from the mechanical port. In both cases, the force input is unknown, but assumed to produce periodic trajectories. To facilitate the derivation of optimal control solutions, fixed levels of average mechanical power $\bar{W}$ transmitted to the load is assumed in the first case, and a fixed level of average electric power ${\bar{S}}_{e}$ transmitted to the source is assumed in the harvesting case, introducing respective equality constraints.

The problem Hamiltonians are required to achieve stationarity with respect to the force input. Remarkably, the resulting feedback controls do not depend on the assumed values of $\bar{W}$ or ${\bar{S}}_{e}$ and do not require force feedback.

**Figure 6:**
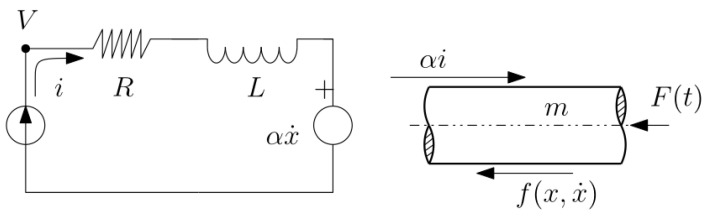
Voice-Coil Actuator Schematic.

Figure 6 shows the physical model, where energy is stored in the inductance *L* and in the mass *m*. The average mechanical and electric energies in [0T] are ${\bar{E}}_{m}=\frac{m}{2T}\oint v^{2}\left(t\right)dt$ and ${\bar{E}}_{e}=\frac{L}{2T}\oint i^{2}dt$, respectively. The instantaneous power transmitted to the load depends on the external force and is given by $\bar{W}=\frac{1}{T}\oint F\left(t\right)\dot{x}\left(t\right)dt$. A possibly nonlinear frictional force $f(x,\dot{x})$ is initially considered. The electromechanical conversion constant is α, defining both the induced voltage $\alpha \dot{x}$ and the induced force αi. Either a current source or a voltage source could have been chosen to analyze power conversion across the interconnected system. However, the former leads to considerable simplification, since the inductor current is an input u≜i and a single state variable, namely the velocity *v*, is necessary in the first-order dynamic model below:

(41) $$\dot{v}=\frac{1}{m}\left(\alpha u-f\left(x,v\right)-F\left(t\right)\right)$$

In addition, Kirchhoff’s law holds for the circuit:

(42) $$L\dot{u}=V-Ru-\alpha v$$

where *V* is the voltage across the current source. The instantaneous power balances per Equation (5) are obtained by multiplying Equation (41) by mv and Equation (42) by *u*, and recognizing the rates of energy for the electrical and mechanical storage elements on the left-hand sides:

(43) $$\begin{array}{ccc} {\dot{E}}_{m} & = & \alpha vu-vf-vF \end{array}$$

(44) $$\begin{array}{ccc} {\dot{E}}_{e} & = & Vu-\alpha vu-Ru^{2} \end{array}$$

### 5.1. Case A: Load Power Transfer Optimization

#### 5.1.1. Subsystem Definition and ECD

A direct and intuitive way to define subsystems from the power balance above and the physical layout would be as follows: $s_{e}=Vu$, $\phi_{me}=\alpha vu$, $\sigma_{m}=vf$, $\sigma_{e}=Ru^{2}$ and w=Fv. However, an abstract definition exists that leads to considerable simplifications. For this, add and subtract $Ru^{2}$ in Equation (43) and add and subtract vf+vF in Equation (44). Define subsystems 1 and 2 with $E_{1}=E_{e}$ and $E_{2}=E_{m}$ so that the power balance to be used becomes

(45) $$\begin{array}{ccc} {\dot{E}}_{2} & = & -\left(vf-Ru^{2}-\alpha vu+vF\right)-Ru^{2} \end{array}$$

(46) $$\begin{array}{ccc} {\dot{E}}_{1} & = & Vu+(vf-Ru^{2}-\alpha vu+vF)-vf-vF \end{array}$$

Under this definition $s_{1}=Vu$, $s_{2}=0$, $\phi_{12}=vf-Ru^{2}-\alpha vu+vF$, $\sigma_{1}=vf$, $\sigma_{2}=Ru^{2}$, $w_{1}=Fv$ and $w_{2}=0$. The rationale for this choice is that subsystem 2 is now cyclopassive with respect to $\phi_{21}$, that is

$${\bar{\phi }}_{21}={\bar{\sigma }}_{2}\geq 0$$

for all cyclic trajectories. The sign-definiteness of $\phi_{21}$ simplifies the ECD condition, which reduces to finding $\beta_{i}$ such that $d_{1}-d_{2}\geq 0$ for all cyclic trajectories. In this case c=0 may be used and we need

(47) $$\frac{{\bar{E}}_{2}}{{\bar{E}}_{1}}\leq \frac{\beta_{1}}{\beta_{2}}$$

Defining $\gamma^{2}≜\beta_{1}/\beta_{2}$, the problem is solved by choosing any $\gamma^{2}$ such that

(48) $$\gamma^{2}\geq \sup\frac{{\bar{E}}_{2}}{{\bar{E}}_{1}}≜\gamma_{crit}^{2}$$

where the supremum is taken over all cyclic trajectories such that ${\bar{E}}_{1}\neq 0$. For linear systems, this has been shown to become a generalized eigenvalue problem [4]. In particular, when ${\bar{E}}_{1}$ depends on the input rather than a state, $\gamma_{crit}^{2}$ may be found from the $H_{\infty }$ norm (The $H_{\infty }$ norm of a transfer function is its peak frequency response magnitude) [36] of the transfer function from the input to the state defining $E_{2}$. To see this, suppose friction is linear with viscous coefficient *b* so that f(x,v)=bv and

$$V\left(s\right)=G_{u}\left(s\right)U\left(s\right)+G_{F}\left(s\right)F\left(s\right)$$

where $G_{u}\left(s\right)=\frac{\alpha }{ms+b}$ and $G_{F}\left(s\right)=-\frac{1}{ms+b}$. Let $\gamma_{crit}^{2}$ be defined as

$$\gamma_{crit}^{2}=\left|\right|G_{u}{\left|\right|}_{\infty }^{2}=\underset{{\bar{E}}_{1}\neq 0}{\sup}\frac{{\bar{E}}_{2}}{{\bar{E}}_{1}}=\frac{m}{L}\underset{{\left||u|\right|}_{2}\neq 0}{\sup}\frac{{\left||v|\right|}_{2}^{2}}{{\left||u|\right|}_{2}^{2}}$$

We claim that no input *F* can yield an energy ratio higher than $\gamma_{crit}^{2}$ above. By contradiction, suppose $\exists u,F\in L_{2}\left[0\;T\right]$ such that $\frac{{\bar{E}}_{2}}{{\bar{E}}_{1}}>\gamma_{crit}^{2}$. Then

$$V\left(s\right)=G_{u}\left(s\right)\tilde{U}\left(s\right)$$

where $\tilde{U}\left(s\right)=U\left(s\right)-F\left(s\right)/\alpha$. But $u,F\in L_{2}\left[0\;T\right]$ implies that $\tilde{u}$ is also in $L_{2}\left[0\;T\right]$, contradicting the definition of $\gamma_{crit}^{2}$. Therefore, for the linear case $\gamma_{crit}^{2}$ becomes $\alpha^{2}/b^{2}$ (here the zero-frequency magnitude of $G_{u}\left(j\omega \right)$).

#### 5.1.2. Objective Function

For this system, the lost work minimization objective takes on a simple form when k=2 is used as a reference. Without loss of generality, take $\beta_{2}=1$ and $\beta_{1}=\gamma^{2}$. Using the 2-norm notation for signals: $d_{1}=\gamma^{2}{\bar{E}}_{1}=\gamma^{2}{L||u||}_{2}^{2}/\left(2T\right)$, $d_{2}={m||v||}_{2}^{2}/\left(2T\right)$ and ${\bar{\sigma }}_{2}=\bar{\phi_{21}}={R||u||}_{2}^{2}/T$. From Equation (39), lost work becomes proportional to

(49) $$d_{2}{\bar{\sigma }}_{2}\frac{d_{1}-d_{2}}{d_{1}d_{2}}\propto \frac{1}{2}\int_{0}^{T}{\hat{\gamma }}^{2}u^{2}-v^{2}\;dt≜J$$

where ${\hat{\gamma }}^{2}=L\gamma^{2}/m$. The ECD condition expressed in terms of ${\hat{\gamma }}^{2}$ is

(50) $${\hat{\gamma }}^{2}\geq \frac{\alpha^{2}}{b^{2}}$$

This cost function is a quadratic sign-indefinite form, similar to those arising in risk-sensitive optimization and differential games.

#### 5.1.3. Optimal Control Problem Formulation

The external force is regarded as unknown, however the problem is solved under the constraint that the average power $\bar{W}$ is specified. The cyclic period *T* is also known and fixed, and periodic boundary conditions are imposed on *x*, *v* and *u*. An additional state *z* is introduced to handle the isoperimetric constraint associated with $\bar{W}$. The OCP becomes

(51) $$\begin{array}{cc} \; & \min_{u\in L_{2}\left[0\;T\right]}\;\;J=\frac{1}{2}\int_{0}^{T}{\hat{\gamma }}^{2}u^{2}-v^{2}\;dt\\ \; & \mathrm{subject}\;\mathrm{to}:\\ \; & \dot{x}=v\\ \; & \dot{v}=\frac{1}{m}\left(\alpha u-f\left(x,v\right)-F\left(t\right)\right)\\ \; & \dot{z}=\frac{1}{T}F\left(t\right)v\\ \; & x(0)=x(T)\\ \; & v(0)=v(T),\;u(0)=u(T)\\ \; & z\left(0\right)=0,\;z\left(T\right)=\bar{W} \end{array}$$

Periodicity of *x* is handled with the additional isoperimetric constraint $\int_{0}^{T}v\left(t\right)dt=0$.

#### 5.1.4. OCP Solution

Note that the force is unspecified, however, a deterministic solution is possible because of the isoperimetric constraint. For this, the standard process of optimal control theory is used, by defining the Hamiltonian

$$H=\frac{1}{2}\left({\hat{\gamma }}^{2}u-v^{2}\right)+\frac{\lambda_{1}}{m}\left(\alpha u-f\left(x,v\right)-F\left(t\right)\right)+\frac{1}{T}\lambda_{2}F\left(t\right)v+\lambda_{3}v$$

where $\lambda_{i}$ are the co-states. The first-order necessary condition for minimization of H with respect to *u* yields the optimal control $u^{*}=-\frac{\alpha \lambda_{1}}{m{\hat{\gamma }}^{2}}$. This is indeed a minimizer, since $\frac{\partial^{2}H}{\partial u^{2}}={\hat{\gamma }}^{2}>0$.

The first-order stationarity condition on input F(t) does not yield a specific function. However, an algebraic relationship between co-states is obtained:

(52) $$T\lambda_{1}=m\lambda_{2}v$$

Next, the co-state equations are obtained from the Hamiltonian:

(53) $$\begin{array}{ccc} {\dot{\lambda }}_{1}=-\frac{\partial H}{\partial v} & = & v+\frac{\lambda_{1}}{m}f_{v}\left(x,v\right)-\frac{\lambda_{2}}{T}F\left(t\right)v-\lambda_{3} \end{array}$$

(54) $$\begin{array}{ccc} \dot{\lambda_{2}}=-\frac{\partial H}{\partial z} & = & 0 \end{array}$$

(55) $$\begin{array}{ccc} \dot{\lambda_{3}}=-\frac{\partial H}{\partial x} & = & \frac{\lambda_{1}}{m}f_{x}\left(x,v\right) \end{array}$$

where $f_{v}\left(x,v\right)=\frac{\partial f(x,v)}{\partial v}$ and $f_{x}\left(x,v\right)=\frac{\partial f(x,v)}{\partial x}$. Then $\lambda_{2}$ is constant and substitution of Equation (52) yields the optimal control

(56) $$u^{*}=-\frac{\alpha \lambda_{2}v}{{\hat{\gamma }}^{2}T}$$

with this, the closed-loop dynamics of velocity become

(57) $$\dot{v}=G\left(v\right)+BF\left(t\right)$$

where $G\left(v\right)=-\frac{1}{m}\left(\frac{\alpha^{2}\lambda_{2}}{{\hat{\gamma }}^{2}T}v+f\left(x,v\right)\right)$ and B=−1/m. The remainder of the study in this section aims to determine the existence of solutions for $\lambda_{2}$ and the resulting system properties. Explicit solution trajectories are only possible for specific instances of F(t). However, much information can be extracted by performing an average power balance on the closed-loop system and a similar operation on the co-state equation for $\lambda_{1}$. Multiply Equation (57) by mv/T and integrate cyclically, eliminating $\lambda_{1}$ from Equation (52) and using the isoperimetric constraint to obtain

(58) $$\bar{W}=-\frac{\alpha^{2}\lambda_{2}}{{\hat{\gamma }}^{2}T^{2}}{\left||v|\right|}_{2}^{2}-\frac{1}{T}\oint_{0}^{T}vf\left(x,v\right)\;dt$$

Similarly, multiply Equation (53) by $\lambda_{1}$, and make substitutions to obtain

$$\bar{W}=\frac{1}{\lambda_{2}}{\left||v|\right|}_{2}^{2}+\frac{1}{T}\oint_{0}^{T}v^{2}f_{v}\left(x,v\right)\;dt-\frac{1}{\lambda_{2}}\oint_{0}^{T}\lambda_{3}v\;dt$$

Combining yields a quadratic equation for $\lambda_{2}$:

(59) $$\frac{\alpha^{2}{\left||v|\right|}_{2}^{2}}{{\hat{\gamma }}^{2}T^{2}}\lambda_{2}^{2}+\left(\frac{1}{T}\oint_{0}^{T}vf\left(x,v\right)+v^{2}f_{v}\left(x,v\right)\;dt\right)\lambda_{2}+{\left||v|\right|}_{2}^{2}-\oint_{0}^{T}\lambda_{3}v\;dt=0$$

If the friction force is independent from *x*, the last term above vanishes from the isoperimetric constraint on *v*. Otherwise, knowledge of f(x,v) would be required in a boundary-value problem to solve the state-costate equations and determine optimal trajectories.

#### 5.1.5. Linear Friction Case

Explicit results are obtained below for viscous friction of the form f(x,v)=bv for some b>0. In this case the quadratic equation for $\lambda_{2}$ reduces to

(60) $$\frac{\alpha^{2}}{{\hat{\gamma }}^{2}T^{2}}\lambda_{2}^{2}+\frac{2b}{T}\lambda_{2}+1=0$$

and the condition for real solutions is ${\hat{\gamma }}^{2}\geq \alpha^{2}/b^{2}$. This condition is the same as required for ECD (Equation (50)) Indeed, maximization of $\bar{E_{2}}/\bar{E_{1}}$ over all cyclic trajectories of Equation (41) such that the average power constraint is met is independent of the value of $\bar{W}$ and matches the square of the $H_{\infty }$ norm of the transfer function from *u* to *v*. The norm is precisely α/b, as shown above.

#### 5.1.6. Solutions for $\lambda_{2}$ vs. Sign of $\bar{W}$ and Stability

For the linear friction case, Equation (58) becomes

$$\bar{W}=-\frac{1}{T}\left(\frac{\alpha^{2}\lambda_{2}}{{\hat{\gamma }}^{2}T}+b\right){\left||v|\right|}_{2}^{2}$$

It can be verified that one of the roots of Equation (60), namely

$$\lambda_{2}=\frac{T{\hat{\gamma }}^{2}}{\alpha^{2}}\left(-b-\sqrt{b^{2}-\frac{\alpha^{2}}{{\hat{\gamma }}^{2}}}\right)$$

must be chosen for $\bar{W}\geq 0$, while the other root corresponds to $\bar{W}<0$. The non-negative average power can then be written as

(61) $$\bar{W}=\frac{{\left||v|\right|}_{2}^{2}}{T}\sqrt{b^{2}-\frac{\alpha^{2}}{{\hat{\gamma }}^{2}}}$$

The closed-loop system has $G\left(v\right)=-\frac{1}{m}\left(\frac{\alpha^{2}\lambda_{2}}{{\hat{\gamma }}^{2}T}+b\right)v$, therefore the optimal control for $\bar{W}\geq 0$ does not stabilize the closed-loop system, indicating that load power transfer enhancement and stability are at odds in this case. However, bounded state and control trajectories (i.e., in $L_{2}\left[0\;T\right]$) are ensured because of the boundary conditions and the finite horizon involved. Also, revisiting Assumption 2, Equation (61) shows that

$$\frac{\bar{W}}{d_{2}}=\frac{2}{m}\sqrt{b^{2}-\frac{\alpha^{2}}{{\hat{\gamma }}^{2}}}$$

showing that a finite $\hat{L}$ exists for the closed-loop system.

#### 5.1.7. Efficiency

The efficiency of average power conversion from the electrical to the mechanical port can be obtained from the above solutions. For $\bar{W}\geq 0$, the efficiency η of the average power conversion in the cycle is $\eta =\frac{\bar{W}}{\bar{S}}$, where the average supply is $\bar{S}=\bar{W}+\bar{\sigma_{1}}+\bar{\sigma_{2}}$. In closed-loop, the losses can be obtained by substitution as

(62) $$\begin{array}{ccc} {\bar{\sigma }}_{1} & = & \frac{b}{T}{\left||v|\right|}_{2}^{2} \end{array}$$

(63) $$\begin{array}{ccc} {\bar{\sigma }}_{2} & = & \frac{R}{T}{\left||u|\right|}_{2}^{2}=\frac{R\alpha^{2}\lambda_{2}^{2}}{{\hat{\gamma }}^{4}T^{3}}{\left||v|\right|}_{2}^{2} \end{array}$$

The efficiency becomes

(64) $$\eta =\frac{\sqrt{b^{2}-\frac{\alpha^{2}}{{\hat{\gamma }}^{2}}}}{\sqrt{b^{2}-\frac{\alpha^{2}}{{\hat{\gamma }}^{2}}}+\frac{R}{\alpha^{2}}{\left(b+\sqrt{b^{2}-\frac{\alpha^{2}}{{\hat{\gamma }}^{2}}}\right)}^{2}+b}$$

#### 5.1.8. Maximal Efficiency Under EGM

Several observations are in order: First, calculus shows that η is a monotonically-increasing function of ${\hat{\gamma }}^{2}$ in the interval of validity of ECD given by ${\hat{\gamma }}^{2}\geq \alpha^{2}/b^{2}$. Next:

(65) $$\lim_{{\hat{\gamma }}^{2}\to \infty }\eta =\frac{1}{2}\frac{\alpha^{2}}{\alpha^{2}+2bR}\leq \frac{1}{2}$$

This means that η=1/2 will never be exceeded, even if R=0. In this respect, EGM coincides with the outcome of the Maximum Power Theorem (MPT) in electric circuits, which shares the same sub-maximal efficiency of 50% [37]. The MPT gives the load impedance that results in maximum power transmitted to it, but does not yield maximum efficiency. In the setting of the MPT, maximum efficiency is obtained at zero power transmission in the absence of other constraints. In this paper, if *b* approaches zero, $\gamma^{2}$ must approach *∞* to satisfy ECD. This limiting case produces $\bar{W}=\bar{S}={\bar{\sigma }}_{1}={\bar{\sigma }}_{2}=0$, as in the MPT.

### 5.2. Case B: Energy Harvesting Optimization

#### 5.2.1. Subsystem Definition and ECD

Subsystems are now chosen so that the average power transfer is directed from the mechanical towards the electrical ports. Define subsystems 1 and 2 with $E_{1}=E_{m}$ and $E_{2}=E_{e}$ so that the power balance to be used becomes

(66) $$\begin{array}{ccc} {\dot{E}}_{1} & = & -vF+(-Vu+\alpha vu)-vf+Vu \end{array}$$

(67) $$\begin{array}{ccc} {\dot{E}}_{2} & = & -\left(-Vu+\alpha vu\right)-Ru^{2} \end{array}$$

Under this definition $s_{1}=-vF$, $s_{2}=0$, $\phi_{12}=-Vu+\alpha vu$, $\sigma_{1}=vf$, $\sigma_{2}=Ru^{2}$, $w_{1}=-Vu$ and $w_{2}=0$. This renders subsystem 2 cyclopassive with respect to $\phi_{21}$, that is ${\bar{\phi }}_{21}={\bar{\sigma }}_{2}\geq 0$.

Comparing with Case A, the structure of the equations is the same, with new definitions for $\phi_{12}$, $s_{1}$, $w_{1}$, $E_{1}$ and $E_{2}$ and the same definitions of $\sigma_{1}$ and $\sigma_{2}$. The ECD condition has the average energies switched:

$$\gamma^{2}\geq \underset{{\bar{E}}_{2}\neq 0}{\sup}\frac{{\bar{E}}_{1}}{{\bar{E}}_{2}}=\frac{L}{m}\underset{{\left||v|\right|}_{2}\neq 0}{\sup}\frac{{\left||u|\right|}_{2}^{2}}{{\left||v|\right|}^{2}}≜\gamma_{crit}^{2}$$

To determine $\gamma_{crit}^{2}$ we consider the mapping from inputs (v,V) to outputs (u,F) given by the system dynamics. As above, the transfer function from velocity input to current output in Equation (42) has infinity norm α/R, thus $\gamma_{crit}^{2}=\frac{L}{m}\frac{\alpha^{2}}{R^{2}}$.

#### 5.2.2. Objective Function

The lost work minimization objective takes on a simple form when k=1 is used as a reference. Taking $\beta_{2}=1$ and $\beta_{1}=\gamma^{2}$, algebraic manipulation shows that the objective function becomes

(68) $$J_{h}=\frac{1}{2}\int_{0}^{T}-{\hat{\gamma }}^{2}u^{2}+v^{2}\;dt$$

where $\hat{\gamma^{2}}=\frac{L}{m\gamma^{2}}$. The ECD condition in terms of ${\hat{\gamma }}^{2}$ is therefore

(69) $${\hat{\gamma }}^{2}\leq \frac{R^{2}}{\alpha^{2}}$$

which is equivalent to that of Case A when the dissipation parameter *b* is replaced by *R*, given the reciprocal definitions of ${\hat{\gamma }}^{2}$.

#### 5.2.3. OCP Formulation

The problem is solved under an equality constraint on the average power harvested ${\bar{S}}_{e}$, which must be negative. An expression for ${\bar{S}}_{e}$ is obtained from Equation (42) by solving for Vu and integrating cyclically. Periodic boundary conditions are imposed on *x* and *v* and an additional state *z* is introduced to handle the isoperimetric constraint associated with ${\bar{S}}_{e}$. The OCP becomes

(70) $$\begin{array}{cc} \; & \min_{u\in L_{2}\left[0\;T\right]}\;\;J_{h}=\frac{1}{2}\int_{0}^{T}-{\hat{\gamma }}^{2}u^{2}+v^{2}\;dt\\ \; & \mathrm{subject}\;\mathrm{to}:\\ \; & \dot{x}=v\\ \; & \dot{v}=\frac{1}{m}\left(\alpha u-f\left(x,v\right)-F\left(t\right)\right)\\ \; & \dot{z}=\frac{1}{T}\left(\alpha vu+Ru^{2}\right)\;dt\\ \; & x(0)=x(T)\\ \; & v(0)=v(T),\;u(0)=u(T)\\ \; & z\left(0\right)=0,\;z\left(T\right)={\bar{S}}_{e} \end{array}$$

Periodicity of *x* is handled with the additional isoperimetric constraint $\int_{0}^{T}v\left(t\right)dt=0$.

#### 5.2.4. OCP Solution

The necessary conditions for an optimal control are applied as above and only main aspects of the derivation are mentioned for conciseness. In this case, stationarity with respect to *F* requires all costates to be constant, yielding the optimal control

(71) $$u^{*}=\frac{\alpha \lambda_{2}v}{({\hat{\gamma }}^{2}-2R\lambda_{2}/T)T}$$

where $\lambda_{2}$ is the costate associated with the equality constraint on ${\bar{S}}_{e}$. In this case, it is possible to show that *even with nonlinear friction*, a quadratic equation for $\lambda_{2}$ is obtained:

(72) $$\frac{\alpha^{2}}{{\hat{\gamma }}^{2}T^{2}}\lambda_{2}^{2}-\frac{2R}{T\hat{\gamma^{2}}}\lambda_{2}+1=0$$

The results below, however have been obtained for the linear viscous friction case, f(x,v)=bv.

#### 5.2.5. Solutions for $\lambda_{2}$ vs. Sign of ${\bar{S}}_{e}$ and Stability

The condition for the existence of real solutions to Equation (72) is again coincident with the ECD requirement: ${\hat{\gamma }}^{2}\leq \frac{R^{2}}{\alpha^{2}}$. Under this condition it is straightforward to show that both solutions for $\lambda_{2}$ yield closed-loop stability. Further, only the “+” solution in the quadratic formula is feasible for harvesting (${\bar{S}}_{e}<0$). It can be shown that the feasible, stable solution also satisfies the second-order condition for a Hamiltonian minimum.

#### 5.2.6. Maximal Efficiency

An efficiency formula for the average power conversion from the mechanical to the electrical port under the above optimal control can be readily obtained. In fact, the efficiency is the same as in Equation (64). Thus as $\gamma^{2}$ approaches the ECD critical value, the efficiency approaches the same maximal limit as in Case A (Equation (65)). EGM coincides with the outcome of the MPT also for the harvesting case.

### 5.3. Comparison with Efficiency Maximization

When either $\bar{W}$ or ${\bar{S}}_{e}$ are preset, efficiency maximization is equivalent to loss minimization. For each of cases A and B, OCP can be formulated using the objective function

$$J_{\eta }=\frac{1}{2}\int_{0}^{T}\sigma_{1}+\sigma_{2}\;dt$$

and subject to the pertinent isoperimetric constraints and boundary conditions. The solution follows standard optimal control techniques as above and is omitted for conciseness. In each case, a quadratic equation is obtained for the co-state $\lambda_{2}$ associated with the isoperimetric constraint. For Case A, the root corresponding to $\bar{W}\geq 0$ is non-stabilizing and the corresponding effiency $\eta_{LM}$ can be obtained. It can be shown that $\eta_{LM}\geq \eta$ regardless of $\bar{W}$. Like in the MPT, a higher efficiency is obtained by maximizing it directly. However, the average power transmitted to the load will be smaller.

The situation is more favorable for harvesting, since closed-loop stability is obtained with the solution for $\lambda_{2}$ that also produces feasibility (${\bar{S}}_{e}<0$). Again, direct loss minimization enhances efficiency, while EGM maximizes the amount of average power harvested.

### 5.4. Summary

Table 1 summarizes all combinations of power directions and objective functions and the solution features in terms of feasibility and stability.

## 6. Numerical Simulation

The results above are examined by applying random periodic forces under the four scenarios of Table 1. The parameters in Table 2 were adopted.

**Figure 7:**
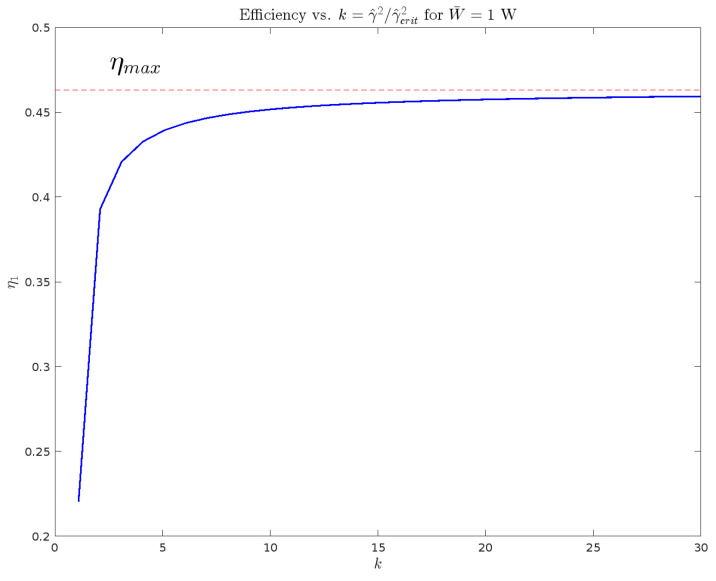
Efficiency with Randomly-Generated Force under Periodicity and Average Load Power Constraints.

The maximal efficiencies for both EGM cases coincide at 46.3% from Equation (65). Instances of F(t) meeting $\bar{W}=1$ W or ${\bar{S}}_{e}=-1$ W were generated by representing the force with a 4-term Fourier series with fundamental period T=2 s. A numerical routine generated random Fourier coefficients under the isoperimetric constraints for the closed-loop system. The resulting force was then applied to the closed-loop system and the velocity trajectories numerically integrated, followed by a calculation of relevant quantities. The efficiencies are plotted in Figure 7 for values of $\gamma^{2}$ ranging from 1.1$\gamma_{crit}^{2}$ to 30$\gamma_{crit}^{2}$.

### Montecarlo Study with Inequality Constraints

To highlight the distinctive features of the EGM OCP solutions against direct loss minimization, the equality constraints were replaced by inequalities. In case A, the average power transmitted to the load was constrained to be at least 1 W. In case B, the average power at the electrical port was constrained to be at most −1 W (an average harvested power of at least 1 W). Monte Carlo simulations were conducted by applying 1000 instances of randomly-generated force to the closed-loop systems in each of the four scenarios in Table 1. A fixed ${\hat{\gamma }}^{2}=30{\hat{\gamma }}_{crit}^{2}$ was used, yielding η=0.459 for the EGM objectives, while $\eta_{LM}=0.672$ applies to the direct loss minimization (LM) cases.

**Figure 8:**
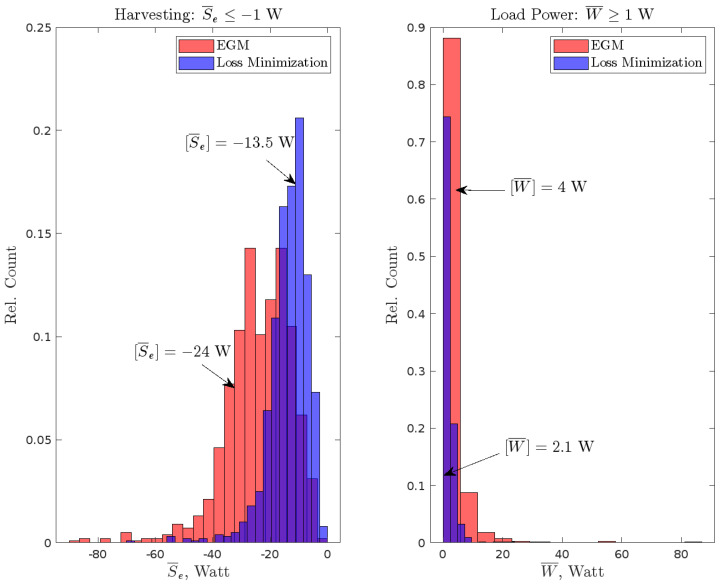
Outcomes with Randomly-Generated Force under Periodicity and Relaxed Average Power Constraints.

Figure 8 shows two sets of normalized histograms side-by-side, with the two harvesting cases on the left and the two load power cases on the right. The results clearly confirm the above observations: the average power transmitted in either direction is statistically higher than that obtained with efficiency maximization. In fact the statistical expectations are $[\bar{W}]=4$ W with EGM vs. 2.1 W with LM (load power); and $[{\bar{S}}_{e}]=-24$ W with EGM vs. −13.5 W with LM (harvesting). It is interesting to note that the distributions of average power are more spread out with EGM and more concentrated with LM. Also, the distributions in the load power cases peak closer to the lower bound of 1 W, while the peaks for harvesting are more distant from the bound of −1 W. More investigation is required to determine the reason for this.

## 7. Discussion

The enhancement of average power transmitted to the mechanical load under both efficiency and EGM criteria is in conflict with stability, as opposite solutions for $\lambda_{2}$ are required. While the results provide information about the limits to power conversion in this direction, the resulting feedback controls are not implementable in practice. Conversely, energy harvesting performance may enhanced according to either criterion while preserving closed-loop stability, since the required solutions for $\lambda_{2}$ coincide. Practical control laws are obtained that require only velocity feedback and offer the best compromise in the absence of information about the applied force. The optimal control laws can be simplified to show that they do not depend on *T*, thus no a-priori information about the force or its spectrum is required in a practical implementation.

It is up to a designer to prioritize efficiency or absolute amounts of harvested power depending on the application. When extracting power from renewable resources, process input comes at no operational cost and can be regarded as inexhaustible. The EGM solution is an attractive option for these applications.

The patterns of stability and direction of average power transmission found in both EGM and LM may not be incidental to the system of the study, but rather and indication of a more fundamental aspect. Further study is required to determine how this applies to more general systems.

A strong correspondence between the features of EGM solutions and the MPT was found, particularly in relation to efficiency maximization through direct minimization of losses.

The study used one of the two possible $d_{k}$ references to obtain the lost work cost function. The choice simplified the problem and made the optimal control gain independent of *R* for the load power case and independent of *b* for the harvesting case. This is yet another attractive feature of the EGM solutions for energy harvesting optimization, due to the usual difficulties associated with friction modeling and identification, particularly in the nonlinear case. Previous work by the authors in energy-optimal motion planning for robots displayed this advantage [32]. The effect of the reference must be studied further. The study may be generalized by considering larger interconnected systems under a linear port-Hamiltonian representation. The methodological steps used here to find a solution through average power balances may also apply. Finally, a mixed, weighted EGM-efficiency criterion is likely to have analytical solutions representing a compromise between both objectives.

## Figures and Tables

**Table 1:** Summary of OCP and solution features. The harvesting case yields consistency between closed-loop stability and feasibility of the desired direction of average power transmission.

	Load Power		Harvesting	
Criterion	EGM	Losses	EGM	Losses
Stability	$\lambda_{2}^{+}$	$\lambda_{2}^{+}$	$\lambda_{2}^{+}$	$\lambda_{2}^{+}$
Obj. Feasibility	$\lambda_{2}^{-}$	$\lambda_{2}^{-}$	$\lambda_{2}^{+}$	$\lambda_{2}^{+}$
Max. Efficiency	1/2	1	1/2	1

**Table 2:** Parameters of the numerical simulation study.

Parameter	Symbol	Value	Units
Mass	*m*	0.1	kg
Inductance	*L*	1	mH
Friction coefficient	*b*	0.1	${\mathrm{Nsm}}^{-1}$
Resistance	*R*	10	Ω
Conversion constant	α	5	${\mathrm{NA}}^{-1}$

## Data Availability

The original contributions presented in this study are included in the article. Further inquiries can be directed to the corresponding author.
